# Determinants of safety climate at primary care level in Ghana, Malawi and Uganda: a cross-sectional study across 138 selected primary healthcare facilities

**DOI:** 10.1186/s12960-021-00617-9

**Published:** 2021-06-07

**Authors:** Frédérique Vallières, Paul Mubiri, Samuel Agyei Agyemang, Samuel Amon, Jana Gerold, Tim Martineau, Ann Nolan, Thomasena O’Byrne, Lifah Sanudi, Freddie Sengooba, Helen Prytherch

**Affiliations:** 1 Trinity Centre for Global Health, Trinity College Dublin, 7-9 Leinster Street South, Dublin 2, Ireland; 2 School of Public Health, Makerere University, Kampala, Uganda; 3 School of Public Health, College of Health Sciences, University of Ghana, Legon, P. O. Box LG13, Accra, Ghana; 4 Swiss Tropical and Public Health Institute, Socinstrasse 57, 4002 Basel, Switzerland; 5 University of Basel, 4003 Basel, Switzerland; 6 Liverpool School of Tropical Medicine, Liverpool, United Kingdom; 7 Research for Equity and Community Health Trust (REACH Trust), Lilongwe, Malawi

**Keywords:** Safety climate, Primary health, Low- and middle-income countries

## Abstract

**Background:**

Safety climate is an essential component of achieving Universal Health Coverage, with several organisational, unit or team-level, and individual health worker factors identified as influencing safety climate. Few studies however, have investigated how these factors contribute to safety climate within health care settings in low- and middle-income countries (LMICs). The current study examines the relationship between key organisational, unit and individual-level factors and safety climate across primary health care centres in Ghana, Malawi and Uganda.

**Methods:**

A cross-sectional, self-administered survey was conducted across 138 primary health care facilities in nine districts across Uganda, Ghana and Malawi. In total, 760 primary health workers completed the questionnaire. The relationships between individual (sex, job satisfaction), unit (teamwork climate, supportive supervision), organisational-level (district managerial support) and safety climate were tested using structural equation modelling (SEM) procedures. Post hoc analyses were also carried out to explore these relationships within each country.

**Results:**

Our model including all countries explained 55% of the variance in safety climate. In this model, safety climate was most strongly associated with teamwork (*β* = 0.56, *p* < 0.001), supportive supervision (*β* = 0.34, *p* < 0.001), and district managerial support (*β* = 0.29, *p* < 0.001). In Ghana, safety climate was positively associated with job satisfaction (*β* = 0.30, *p* < 0.05), teamwork (*β* = 0.46, *p* < 0.001), and supportive supervision (*β* = 0.21, *p* < 0.05), whereby the model explained 43% of the variance in safety climate. In Uganda, the total variance explained by the model was 64%, with teamwork (*β* = 0.56, *p* < 0.001), supportive supervision (*β* = 0.43, *p* < 0.001), and perceived district managerial support (*β* = 0.35, *p* < 0.001) all found to be positively associated with climate. In Malawi, the total variance explained by the model was 63%, with teamwork (*β* = 0.39, *p* = 0.005) and supportive supervision (*β* = 0.27, *p* = 0.023) significantly and positively associated with safety climate.

**Discussion/conclusions:**

Our findings highlight the importance of unit-level factors—and in specific, teamwork and supportive supervision—as particularly important contributors to perceptions of safety climate among primary health workers in LMICs. Implications for practice are discussed.

## Background

Recent decades have seen progress towards Universal Health Coverage (UHC) across many low- or middle-income countries (LMICs), mostly through an expansion of basic health services and strengthening of primary health care. Changing health needs and growing expectations from residents of LMICs however, suggests that increasing physical and financial access to, and coverage of, health care remains insufficient, with a need to also improve the quality of existing health systems [1]. Consequently, increasing attention has been given to the importance of balancing efficiency of delivery, with the need to deliver high-quality, safe patient care.

Safety culture, as one aspect of an organisation’s culture, has been defined as “the product of individual and group values, attitudes, perceptions, competencies, and patterns of behaviour that determine the commitment to, and the style and proficiency of, an organisation’s health and safety management” (p.1) [2]. Furthermore, Sexton et al. [3] indicate that when examining group-level perceptions, the most appropriate term to use is *climate* (e.g., safety climate, or teamwork climate), in reference to the more readily measurable aspects of safety culture, and as opposed to other aspects of culture such as behaviour and values. In this way, safety climate acts as an emergent property; a social construct, characterising groups of individuals based on their shared perceptions of enacted policies and practices that serve as an indicator of the true priority of safety against other organisational goals [4].

The factors that influence safety climate are, by extension, multiple and complex, with an increasing amount of literature focused on identifying its various antecedents, moderators, mediators, and outcomes [5]. Yu and Liang [6], for example, highlight a number of indicators of safety climate, including working conditions, teamwork climate, job satisfaction, stress recognition, and perceptions of management. Likewise, Vincent and colleagues [7] describe several factors that influence safety and quality in clinical practice according to *organisational-level* factors such as staffing levels, workplace and managerial support; *unit or team-level* factors such as teamwork and supervision; as well as *individual-level factors* such as health worker job satisfaction and perceptions of management.

### Organisational-level factors

Alsalem et al. [8] reinforce that safety climate refers to the ability of healthcare organisations to learn effectively from adverse events and implement preventative measures to reduce related harm to patients. At an organisational level, managerial safety practices have been found to mediate the relationship between safety procedures/information flows and clinical incidents in Sweden [9], with quality management systems emerging as an important correlate of both perceived teamwork and safety climate within European hospitals [10]. Within LMICs, and particularly within decentralised contexts, managerial responsibilities tend to be held within district-level health management teams (DHMTs), who oversee the delivery of services (i.e. planning, budgeting, fundraising, monitoring); management of health workforce and other resources, including through paying attention to quality and safety; as well as stakeholder coordination (partners, citizens, patients and other service-users, etc.). Managerial support is therefore considered particularly important to ensure that primary health care centres have all of the resources required for system performance and the delivery of quality health services in LMICs [11].

### Unit-level factors

At unit or team-level, factors such as supervision, and more specifically supportive supervision [12, 13], is widely recognised as important for the improvement of service quality across a range of primary health care services [14–17]. Previous research suggests a strong positive relationship between safety climate and occupational safety at the unit-level [18, 19], in support of group-level factors as an important determinant of climate safety. Likewise, good collaboration with co-workers and an environment that encouraged safety reporting were found to be positively associated with safety attitudes [20]. Consequently, team training and other unit-based strategies that promote teamwork processes, such as cooperation, open communication, and leadership, are often seen as a key strategy to improve the work environment and patient safety [19].

### Individual factors

Finally, among individual factors, previous research report that cadre plays a role, with attitudes towards patient safety differing across doctors, nurses, and allied health professionals, while attention is also called to variations according to the culture in the country of training [21–23]. Age has been identified as an important determinant of safety climate, with older staff expressing more favourable safety attitudes [23]. Likewise, individual health worker knowledge, motivation [24], and job satisfaction have been associated with health worker perceptions of safety climate [25].

Taken together, there is broad agreement that the concepts of both quality and safety need to be investigated within the contexts and systems within which errors and adverse events occur [8]. While patient safety has been widely explored within hospital settings however, fewer studies to date have focused on patient safety within primary health care settings [26]. Moreover, most of the research examining determinants of safety climate within primary care settings to date has taken place in higher income countries, including the upper-middle-income countries of Iran [27] and Brazil [28]. Finally, and given the strong cultural component to safety climate, with very practical implications for managers and practitioners dealing with multi-national organisational contexts, there is a need for more research examining safety-related perceptions across non-Western settings [29]. In light of these gaps, the current study sought to examine the relationship between key organisational, team and individual-level factors and safety climate across primary health care centres in Ghana, Malawi and Uganda.

## Methods

The current baseline research took place within the broader remit of the PERFORM2Scale (https://www.perform2scale.org) programme, as part of regular programme evaluation. Drawing on previous work [30, 31], PERFORM2Scale facilitates the implementation of a management strengthening intervention (MSI) process with district health managers, as those responsible for the managerial oversight of primary care level health workforce.

### Study design

This study is based on a cross-sectional, self-report survey conducted in 138 health care facilities across nine districts: three districts in Uganda (October 2018), three districts in Malawi (November–December 2018), and three districts in Ghana (February–March 2018). All public primary care facilities within the nine selected districts were included across the three countries, with the exception of private and non-governmental organisation (NGO) facilities; as these are not under the full jurisdiction of the DHMTs. Table 1 summarises the characteristics of the health facilities surveyed within each district.

**Table 1:** Primary health care facilities sampled within each country

Country	Districts	Health facilities*	Total health facilities
Ghana	Fanteakwa	Community health and planning services (CHPS) (*n* = 6) Health centres (*n* = 3) Faith-based health centre (*n* = 1) Hospital (*n* = 1)	11
	Suhum	CHPS (*n* = 11) Health centres (*n* = 5) Hospitals (*n* = 1)	17
	Yilo Krobo	Health centres (*n* = 5) Polyclinics (*n* = 2)	7
Uganda	Luwero	Level IV (*n* = 5), Level III (*n* = 13) Level II (*n* = 9)	27
	Wakiso	Hospital (*n* = 1) Level IV (*n* = 5) Level III (*n* = 22)	28
	Nakaseke	Hospital (*n* = 1) Level IV (*n* = 2) Level III (*n* = 7) Level II (*n* = 9)	19
Malawi	Dowa	Hospital (*n* = 1) Health centres (*n* = 11)	12
	Ntchisi	Hospital (*n* = 1) Health centres (*n* = 6)	7
	Salima	Hospital (*n* = 1) Health centres (*n* = 9)	10
Total			138

*In Ghana, CHPS offer community health activities and basic out-patient care services; health centres are the main providers of primary health care, offering out-patient care services, laboratory services, antenatal care and basic obstetric and postpartum services; hospitals and polyclinics offer all services provided by health centres in addition to comprehensive emergency obstetric and newborn care services; blood transfusion and operative care. In Uganda, Level II health centres provide basic out-patient care services, Level III provide antenatal care and basic emergency obstetric care and postpartum services, and Level IV offer all services provided at Level III in addition to operative care and laboratory services. Hospitals provide comprehensive emergency obstetric and newborn care services, blood transfusion, and laboratory services. In Malawi, health centres provide basic out-patient primary care services, whereas hospitals offer both inpatient and outpatient care, often acting as referral centres for health centres. Staffing wise, more rural health centres would consist of a medical assistant, nurses, health surveillance assistants, and environmental health officers, whereas hospitals would include cadres spanning health surveillance officers to specialists

### Study population

Across all three countries, participants were health workers currently employed within a health facility offering primary health care services. Employment covered both frontline staff and facility managers. The sampling of study participants varied slightly between countries, corresponding to the actual numbers of health workers employed in the identified three districts in each country.

In Ghana, the sample size was determined based on a published sample size table (Israel, 2009), with an estimated number of clinical health staff (i.e. professional groups) within the three districts estimated at 600, a precision level of ± 5%, confidence level of 95% and degree of variability of 0.5, and a potential non-response rate of 5%, resulting in a sample size of 252 health workers. Ultimately, 241 participants were recruited (*n* = 182, 75.5% female; *n* = 59, 24.5% male). In Uganda, all technical health workers including health facility management that were present at the health facility and/or hospital on day of data collection, were invited to participate in the study. Call-backs were made to facilities with high numbers of staff, but poor attendance on the day of data collection. A total of 466 responses (*n* = 326, 70% female; *n* = 140, 30% male) were collected across the three districts in Uganda.

In Malawi, health workers eligible from the district hospital and government facilities were listed for each district and then sampled proportion to size of each facility’s health workforce. This resulted in a total number of 67 health workers eligible for the survey drawn from 29 facilities across the three districts. To allow for potential non-response, an additional 20% was added, for a total of 80 health workers. A total of 53 health workers (*n* = 22, 41.5% female; *n* = 31, 58.5% male) across 29 health facilities, including district hospitals, were ultimately included. In total, 760 health workers, of which 30.3% (*n* = 230) were male and 69.7% (*n* = 530) were female, completed the questionnaire across all three countries.

### Data collection

In-country members of the PERFORM2Scale project distributed a self-administered, paper-based health worker questionnaire to health workers. Written informed consent was obtained from all study participants and all surveys were completed in English.

### The health worker questionnaire

The questionnaire included 50 closed-ended items asking about the health workers’ socio-demographic characteristics, including sex (coded 0 = males, 1 = females), country (coded 0 = Uganda, 1 = Ghana, 2 = Malawi), type of health cadre (i.e. professional title), health centre level (where applicable), as well as their perception of safety climate, teamwork climate, supportive supervision, job satisfaction, and district managerial support. The questionnaire was piloted in all countries, with each country’s version undergoing slight language modifications to better suit the individual context, based on feedback received. Those health workers who piloted the questionnaire were not part of the study population.

Safety Climate and Teamwork Climate were measured using the respective subscales from Sexton et al.’s (2006) validated Safety Attitudes Questionnaire—Short Form (SAQ). All items were rated using a five-point Likert scale, anchored by *Strongly Disagree* (= 1) and *Strongly Agree* (= 5). The six-item Teamwork Climate sub-scale was designed to capture the perceived quality of collaboration between health workers, whereas the six-item Safety Climate sub-scale was designed to capture perceptions of a strong and proactive organisational commitment to safety. The SAQ has previously been found to have good psychometric properties [32–34], with both the teamwork climate and safety climate subscales found to have acceptable internal reliability in the current sample (*α* = 0.77, *α* =, *α* = 0.72, respectively).

Perceived Supervision was measured using the validated Perceived Supervision Scale (PSS) [35], a six-item scale scored on a five-point Likert-type scale again ranging from *Strongly Disagree* (= 1) to *Strongly Agree* (= 5). The PSS was chosen as it has been widely used across LMICs [13, 36] and was found to have good internal reliability in the current sample (*a* = 0.87).

Job satisfaction was measured using Warr et al.’s (1979) 10-item Job Satisfaction Scale. The job satisfaction scale was chosen as it has been widely used within medical practitioner research, and has been validated for use among clinicians [37]. Items on the job satisfaction scale were scored on a five-point Likert-type scale ranging from *Very Dissatisfied* (= 1) to *Very Satisfied* (= 5), whereby participants answer in terms of ‘How satisfied or dissatisfied they were’ with a number of extrinsic and intrinsic job-related items. The job satisfaction scale was found to have good internal reliability in the current sample (*α* = 0.84).

District managerial support was assessed using a newly developed set of eight items, whereby participants answer in terms of ‘how much they agree’ with a number of related items. Answers are scored on a five-point Likert-type scale ranging from *Strongly Agree* (= 1) to *Strongly Disagree* (= 5). District managerial support was found to have good internal reliability in the current sample (*α* = 0.77).

All variables were chosen based on the review of extant literature, and guided by the framework put forward by Vincent et al. [7], ensuring at least one variable at the individual, unit and organisational levels.

### Data analysis

**Fig. 1:**
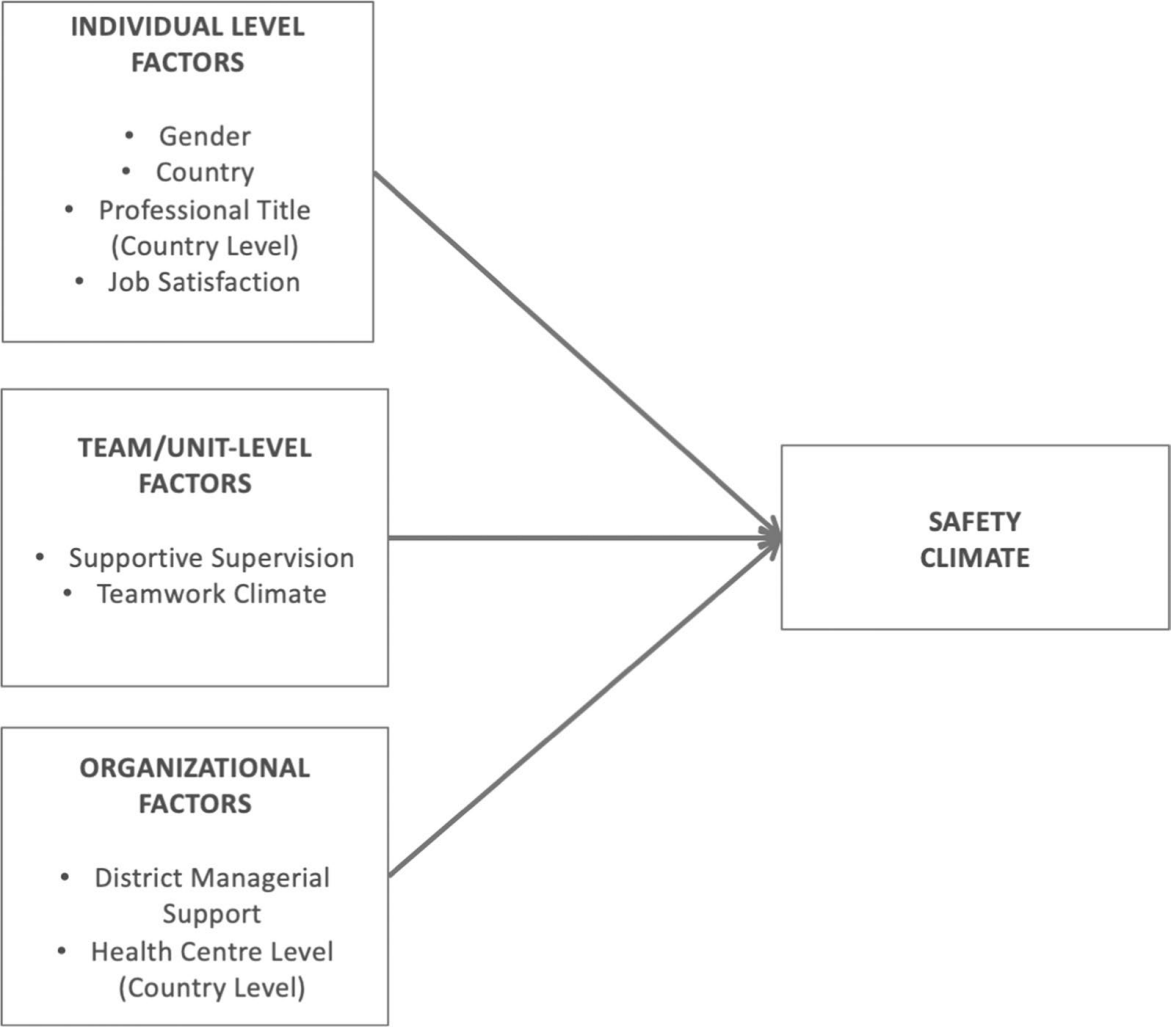
Study’s analytical framework

The relationships between organisational, team, individual factors and safety climate, as outlined the study’s theoretical framework (see Fig. 1), were tested using structural equation modelling (SEM) procedures. SEM was chosen for its ability to confirm the psychometric properties of the measurements employed as well as the relationships between the latent variables [38], while correcting for measurement error as well as testing the strength of the model in explaining the observed pattern of data [39].

First, confirmatory factor analysis (CFA) was used to assess the factor structure of the individual scales. Optimal fit indicators were sought and therefore items demonstrating poor loadings (< 0.3) were removed. Measurement model goodness of fit was assessed using a number of widely recognised fit indices [40, 41] including: a non-significant Chi-square (*χ*^*2*^), Comparative Fit Index (CFI:42) and Tucker–Lewis Index (TLI: 43) values above 0.95 reflect excellent fit, while values above 0.90 reflect acceptable fit; Root-Mean-Square Error of Approximation with 90% confidence intervals (RMSEA 90% CI 44), and Standardised Root-Mean-Square Residual (SRMR: 45) values of 0.06 or less reflect excellent fit while values less than 0.08 reflect acceptable fit. For the models based on Robust Maximum Likelihood estimator (MLR) estimation, the Bayesian Information Criterion (BIC: 46) was used to evaluate and compare models, with the smallest value indicating the best fitting model. Second, a structural analysis, was used to determine the effects of organisational, team and individual-level factors on safety climate [47]. Data analyses were carried out using STATA (Version 15) and SPSS (Version 26). In the structural phase, individual factors (job satisfaction, country, sex), team (supportive supervision, teamwork), and organisational (perceived district managerial support) were sequentially added into the model to determine their added contribution in explaining variations in safety climate.

## Results

### Measurement phase

Table 2 presents the descriptive statistics for each variable, across the various countries. All five scales demonstrated acceptable model fit during the measurement modelling phase (see Table 3).

**Table 2:** Variable descriptives by country

Variable	Country	*N* = 760	Mean	SD
Teamwork climate	Ghana	239	3.82	0.72
	Malawi	53	3.65	0.84
	Uganda	465	3.75	0.66
	**All**	**757**	**3.77**	**0.69**
District managerial support	Ghana	235	3.65	0.61
	Malawi	51	3.31	0.85
	Uganda	461	3.59	0.67
	**All**	**747**	**3.59**	**0.67**
Supportive supervision	Ghana	239	3.89	0.78
	Malawi	51	3.46	1.11
	Uganda	464	3.88	0.74
	**All**	**754**	**3.86**	**0.79**
Job Satisfaction	Ghana	235	3.54	0.55
	Malawi	53	3.34	0.83
	Uganda	465	3.46	0.67
	**All**	**753**	**3.48**	**0.65**
Safety climate	Ghana	239	3.70	0.58
	Malawi	53	3.45	0.69
	Uganda	462	3.70	0.59
	**All**	**754**	**3.68**	**0.60**

**Table 3:** Model fit statistics for each of the study scale

Scales	No. items	*χ*^2^	df	*p*	CFI	TLI	RMSEA (90% CI)	SRMR	BIC
Teamwork climate (Sexton et al. 2006)	6	14.1	8	0.080	0.99	0.98	0.032 (0.001–0.058)	0.020	12,139.5
District managerial support	8	415.4	113	0.000	0.93	0.92	0.059 (0.050–0.083)	0.043	31,295.8
Perceived Supervision (Vallières et al. 2018)	5	2.9	4	0.571	1.0	1.0	0.00 (0.000–0.049)	0.007	8057.6
Job satisfaction Scale (Warr et al. 1979)	10	128.3	31	0.000	0.95	0.93	0.064 (0.053–0.076)	0.036	19,550.3
Safety Climate (Sexton et al. 2006)	5	5.6	4	0.230	0.99	0.99	0.023 (0.000–0.063)	0.013	9856.3

*χ*^2^ Chi-square goodness of fit statistic, *df* degrees of freedom, *p* statistical significance, *CFI* Comparative Fit Index, *TLI* Tucker–Lewis Index, *RMSEA (90% CI)* root-mean-square error of approximation with 90% confidence intervals, *SRMR* standardised square root mean residual, *BIC* Bayesian Information Criterion

### Structural phase

Results for all countries combined are presented in Table 4.

**Table 4:** Structural model results for all countries combined

Covariate	Model 1: individual-level factors		Model 2: individual-level + unit-level factors		Model 3: individual-level + unit + organisational-level factors	
	*β*	*p*-value	*β*	*p*-value	*β*	*p*-value
Sex (ref)	− 0.011	0.762	− 0.01	0.786	− 0.01	0.889
Country						
Uganda (ref)	1		1		1	
Ghana	− 0.09	0.014*	− 0.08	0.044	− 0.09	0.033
Malawi	− 0.17	< 0.001**	− 0.13	0.002**	− 0.12	0.005**
Job satisfaction	0.48	< 0.001**	0.19	< 0.001**	0.09	0.131
Teamwork climate			0.59	< 0.001**	0.56	< 0.001**
Supportive supervision			0.37	< 0.001**	0.34	< 0.001**
District managerial support					0.29	< 0.001**
*R*^2^	0.26		0.54		0.55	

*p < 0.05, **p < 0.01 denote statistically significant association (bold). *β* denotes the standardised coefficient

In the first step, individual factors explained 26% of the variance for safety climate. More specifically, job satisfaction (*β* = 0.48, *p* < 0.001) significantly predicted safety climate and countries differed significantly in their perception of safety climate.

Step two of the model, which included unit-level factors, revealed an additional 28% of the variance in safety climate. In this step teamwork (*β* = 0.59, *p* < 0.001) and supportive supervision (*β* = 0.37, *p* < 0.001) emerged as the strongest predictors of safety climate. Country and job satisfaction also remained statistically significant (*β* = 0.19, *p* < 0.001). The final step, which included organisational factors, revealed an additional 1% of the variance in safety climate. Overall, at step three the total variance explained by the model was 55%. In this model, teamwork (*β* = 0.56, *p* < 0.001) and supportive supervision (*β* = 0.34, *p* < 0.001) remained highly significant contributors to safety climate variance. In addition, district managerial support also emerged as significant (*β* = 0.29, *p* < 0.001). Given the noted effect for country, a series of post hoc analyses were carried out for each country, including type of health worker cadre and health centre level as additional individual-level and organisational factors in the model, respectively. While a structural equation model was tested for Uganda and Ghana, a hierarchical regression model was applied to the Malawi data, given the relatively low sample size. The health centre-level variable was also excluded from the Malawi analysis given that there was only one hospital, as one of the two categories (hospital vs. health centre). Results by country are presented in Table 5.

**Table 5:** Results by country

Covariate	Model 1: individual-level factors		Model 2: individual-level + unit-level factors		Model 3: individual-level + unit + organisational-level factors	
	*β*	*p*-value	*β*	*p*-value	*β*	*p*-value
Ghana						
Male	0.069	0.314	0.012	0.878	0.005	0.947
Job satisfaction	0.65	< 0.001**	0.381	< 0.001**	0.304	0.011*
Health worker qualification						
Administration (Ref)	1		1		1	
Clinical	0.233	0.011*	0.203	0.050	0.115	0.322
Public health staff	0.075	0.439	0.006	0.951	− 0.119	0.336
Team work			0.453	< 0.001**	0.459	< 0.001**
Supportive supervision			0.201	0.049*	0.210	0.043*
District managerial support					0.130	0.243
Level of health facility						
Community based					1	
District hospital					− 0.168	0.168
Health centre					− 0.024	0.828
*R*^2^	0.46		0.43		0.43	
Uganda						
Male	0.034	0.524	0.023	0.629	0.027	0.575
Health worker qualification						
Non-clinical (Ref)	1		1		1	
Clinical	− 0.052	0.329	0.006	0.896	0.191	0.693
Job satisfaction	0.343	< 0.001**	0.149	0.005*	0.073	0.208
Team work			0.607	< 0.001**	0.563	< 0.001**
Supportive supervision			0.468	< 0.001**	0.432	< 0.001**
District managerial support					0.348	< 0.001**
Health facility level						
HC II					− 0.002	0.968
HC III					0.095	0.143
HC IV					0.028	0.650
Hospital					1	
*R*^2^	0.12		0.61		0.64	
Malawi						
Male	0.25	0.847	0.053	0.606	0.041	0.699
Health worker qualification						
Non-clinical (Ref)						
Clinical	0.216	0.109	0.198	0.074	0.205	0.068
Job satisfaction	0.689	< 0.001**	0.260	0.042*	0.223	0.107
Teamwork			0.423	0.002*	0.394	0.005*
Supportive supervision			0.292	0.012*	0.272	0.023*
District managerial support					0.087	0.491
*R*^2^	0.46		0.63		0.63	

*p < 0.05, **p < 0.01 denote statistically significant associations (bold). *β* denotes the standardised coefficient

### Ghana

In the first step, individual factors explained 46% of the variance for safety climate. Job satisfaction (*β* = 0.65, *p* < 0.001) and being clinical staff (compared to administrative staff; *β* = 0.23, *p* < 0.05) significantly predicted higher levels of safety climate. Step two of the model, which included unit-level factors, explained a reduced 43% of the variance in safety climate. In this step, teamwork (*β* = 0.45, *p* < 0.001), job satisfaction (*β* = 0.38, *p* < 0.001), and supportive supervision (*β* = 0.20, *p* < 0.05) emerged as the strongest predictors of safety climate. The final step, which included organisational factors, did not explain any additional variance in safety climate. Overall, at step three the total variance explained by the model was 43%. In this model, job satisfaction (*β* = 0.30, *p* < 0.05), teamwork (*β* = 0.46, *p* < 0.001) and supportive supervision (*β* = 0.21, *p* < 0.05) remained significant contributors to safety climate variance.

### Uganda

In the first step, individual factors explained 12% of the variance for safety climate, with only job satisfaction (*β* = 0.34, *p* < 0.001) found to predict safety climate. Step two of the model, explained an increased 61% of the variance in safety climate. In this step, job satisfaction (*β* = 0.15, *p* = 0.005), teamwork (*β* = 0.61, *p* < 0.001), and supportive supervision (*β* = 0.47, *p* < 0.001) emerging as strong predictors of safety climate. The final step, which included organisational factors, explained an additional 3% of variance in safety climate. Overall, at step three the total variance explained by the model was 64%. In this model, teamwork (*β* = 0.56, *p* < 0.001) and supportive supervision (*β* = 0.43, *p* < 0.001) remained highly significant contributors to safety climate variance. In addition, district managerial support (*β* = 0.35, *p* < 0.001) was also found to predict safety climate.

### Malawi

Each step of the hierarchical regression model was significant (*p* < 0.001). In the first step, individual factors significantly contributed to the model *F* [3, 45] = 12.51, *p* < 0.001. Individual factors explained 46% of the variance for safety climate, with only job satisfaction (*β* = 0.69, *p* < 0.001) associated with safety climate. Step two of the model, which included unit-level factors, explained up to 63% of the variance in safety climate [*F* (5, 43) = 17.52, *p* < 0.001]. In this step, job satisfaction (*β* = 0.26, *p* = 0.042), teamwork (*β* = 0.42, *p* = 0.002), and supportive supervision (*β* = 0.29, *p* = 0.012) emerged as strong predictors of safety climate. The final step, which included organisational factors did not explain any additional variance in safety climate. Overall, at step three the total variance explained by the model was 63%, *F* (5, 43) = 14.5, *p* < 0.001). In this model, teamwork (*β* = 0.39, *p* = 0.005) and supportive supervision (*β* = 0.27, *p* = 0.023) remained significant contributors to safety climate variance. District managerial support was not found to predict safety climate.

## Discussion

The current study sought to examine the relationship between key organisational, team and individual-level factors and safety climate, as central to ensuring quality health care and achieving UHC, across 138 selected primary health care centres in Ghana, Malawi and Uganda. Overall, results across all three countries suggest that unit-level factors, and more specifically, teamwork climate and supportive supervision, emerge as factors that best account for the variance in safety climate in the current samples. This finding is consistent with previous studies that emphasise the importance of supportive supervision [14–17] and teamwork [20] as important correlates of service quality and patient safety across a range of primary health care services [19, 48]. Zaheer et al. [49], for example, also found that teamwork and perceptions of leadership (at supervisory level) were positively associated with perceptions of patient safety among Canadian nurses and allied health professionals. Likewise, Kristensen et al. found positive associations between the implementation of quality management systems and both teamwork and safety climate among over 8500 clinical leaders and frontline clinicians sampled across seven European countries [10]. A positive association between safety climate and teamwork was also reported in a sample of Jordanian nurses [50]. More recently, teamwork and organisational learning was also highlighted in a facility based study in Ethiopia, where patient safety culture was significantly associated with reporting of adverse events including an exchange of feedback about errors [51]. Accordingly, one possible mechanism through which teamwork may facilitate safety climate is through the adoption of practices such as quality improvement.

The important role of supportive supervision, including a joint problem-solving focus, the sense of joint responsibilities and teamwork, cross-learning and skill sharing, as well as the facilitating and coaching role of the supervisor, has been reported among health workers in other African contexts [13]. As part of a supportive environment that fosters strong teamwork and supportive approaches, supervision is likely more conducive to health workers learning from their mistakes, allowing for course correction, and reducing repeated errors. In contrast, more punitive systems—or supervisory approaches that focus on fault-finding, inspection, and control [52]—increases the risk that blame might be apportioned, thus incentivising health workers to hide, cover-up or not admit to errors or mistakes [53, 54].

Individual country analyses suggest variations in whether or not perceived district-level support was associated with perceived safety climate across contexts. Where no association was found, it is possible that support at facility level compensated for the absence of more senior (i.e. district level) managerial support [49]. In Malawi, for example, supportive supervision is sometimes associated with development partners, rather than DHMTs, which may explain why managerial support is not necessarily associated to the district. Moreover, in Ghana for example, while DHMTs offer technical and administrative support to primary healthcare units, the DHMT maintain narrow decision-space for human resource and fiscal decentralisation [55]. Increased decision-making power of sub-districts or unit heads regarding task shifting and task sharing could enhance teamwork, thereby improving safety climate for PHC service delivery in Ghana [56]. Additionally, positive associations may be explained by closer interactions between district managers and primary care facilities, as conducive to shaping a good working environment and conditions within primary health care facilities. Specifically, health workers receiving supportive district managerial support may have more avenues to voice their grievances and challenges they face, and may receive more support in terms of supervision, resources and equipment, all of which are necessary for greater workplace safety climate [48, 57, 58]. Indeed, the responsibility for inspiring teamwork, motivation, providing supportive supervision, and fostering positive staff attitudes is still widely seen as falling under the remit of the DHMTs [59]. For example, in Malawi, the integrated supportive supervision system (ISS) is widely used by DHMTs across the country, as part of their Service Delivery Integration-Systems (SSDI-Systems). In Ghana, the observation that job satisfaction remained positively associated with safety climate, while also accounting for the unit-level factors of teamwork and supportive supervision, is consistent with previous studies, demonstrating that perceptions of safety climate can also influence job satisfaction, whereby employees who report high levels of perceived safety climate also report high levels of job satisfaction [60].

The current study is not without limitations. Firstly, the cross-sectional nature of our study design does not allow for inferences of causality. While teamwork and supportive supervision may contribute towards better safety climate, it is also likely that within health facilities with positive or favourable safety climate, health workers are more likely to work as a team and have supervision mechanisms in place encouraging them to perform their duties to a high standard. More likely however, the relationships between teamwork, supportive supervision, and climate safety are likely multidirectional and mutually reinforcing, rather than unidirectional in nature. Second, and as safety climate was measured using a self-report measure, as an indicator of *perceived* safety climate, we cannot reliably ascertain whether higher levels of unit-level factors are associated with more objective accounts of safety climate. Third, other known correlates of climate safety, such as work engagement, safety behaviour, health worker knowledge and motivation [24], availability of resources and equipment, and interpersonal interactions, the latter of which showed the strongest association with safety climate in a meta-analysis also conceptually building on Zohar’s model [61], were not included as part of the original study design. It is possible that these other factors may act as stronger correlates of safety climate. Finally, the different sampling methods used across the three different study locations pose a challenge to comparing results between countries.

## Conclusion

Together, our findings resonate with previous studies conducted by Yu and Liang [6] and Vincent et al.’s framework [7], both of which highlight the importance of teamwork climate, supervision and perceptions of management/managerial support as important contributors to safety climate. In addition, our findings highlight the importance of unit-level factors (teamwork and supportive supervision) as particularly important contributors to perceptions of safety climate among primary health workers in LMICs. Initiatives aiming to improve perceived safety climate within primary health care centres, including planned initiatives within PERFORM2Scale, may want to consider paying particular attention to teamwork and improving supportive supervision practices as key correlates of safety climate.

## Data Availability

Data are available, upon reasonable request, from the first (FV) or second author (PM).

## Abbreviations

BIC: Bayesian Information Criterion
CFI: Comparative Fit Index
DHMT: District health management team
ISS: Integrated supportive supervision
LMIC: Lower-middle income countries
PSS: Perceived supervision scale
RMSEA: Root-mean-square error of approximation
SAQ: Safety attitudes questionnaire
SEM: Structural equation modelling
SRMR: Standardised root-mean-square residual
SSDI-Systems: Service delivery integration-systems
TLI: Tucker–Lewis Index
UHC: Universal Health Coverage
